# SARS-CoV-2 and Influenza Virus Co-Infection Cases Identified through ILI/SARI Sentinel Surveillance: A Pan-India Report

**DOI:** 10.3390/v14030627

**Published:** 2022-03-17

**Authors:** Neeraj Aggarwal, Varsha Potdar, Neetu Vijay, Labanya Mukhopadhyay, Biswajyoti Borkakoty, S. Manjusree, Manohar Lal Choudhary, Deepika Chowdhury, Riya Verma, Sumit Dutt Bhardwaj, Neelanjana Sarmah, Sreelatha K. H., Prabhat Kumar, Nivedita Gupta

**Affiliations:** 1 Virology Unit, Division of Epidemiology and Communicable Diseases, Indian Council of Medical Research, New Delhi 110029, India; aggarwal.n@icmr.gov.in (N.A.); drneetuvijay@gmail.com (N.V.); labanya.mukhopadhyay@gmail.com (L.M.); 2 National Institute of Virology, Pune 411001, India; potdarvarsha9@gmail.com (V.P.); mlchoudhary@gmail.com (M.L.C.); deepika.pune@gmail.com (D.C.); riyarebeccaverma@gmail.com (R.V.); bhardwaj.sumit@nic.in (S.D.B.); 3 Regional Medical Research Center, Dibrugarh 786010, India; biswaborkakoty@gmail.com (B.B.); sarmahneelanjana@gmail.com (N.S.); 4 Virus Research and Diagnostic Laboratory, Government Medical College, Thiruvananthapuram 695011, India; dr_manjusree@yahoo.co.in (S.M.); sreelathakh@gmail.com (S.K.H.); 5 Division of Biomedical Informatics, Indian Council of Medical Research, New Delhi 110029, India; prabhatchauhan616@outlook.com

**Keywords:** SARS-CoV-2, COVID-19, influenza, co-infection, surveillance

## Abstract

SARS-CoV-2/influenza virus co-infection studies have focused on hospitalized patients who usually had grave sequelae. Here, we report SARS-CoV-2/influenza virus co-infection cases from both community and hospital settings reported through integrated ILI/SARI (Influenza Like Illness/Severe Acute Respiratory Infection) sentinel surveillance established by the Indian Council of Medical Research. We describe the disease progression and outcomes in these cases. Out of 13,467 samples tested from 4 July 2021–31 January 2022, only 5 (0.04%) were of SARS-CoV-2/influenza virus co-infection from 3 different sites in distinct geographic regions. Of these, three patients with extremes of age required hospital admission, but none required ICU admission or mechanical ventilation. No mortality was reported. The other two co-infection cases from community settings were managed at home. This is the first report on SARS-CoV-2/Influenza virus co-infection from community as well as hospital settings in India and shows that influenza viruses are circulating in the community even during COVID-19. The results emphasize the need for continuous surveillance for multiple respiratory pathogens for effective public health management of ILI/SARI cases in line with the WHO (World Health Organization) recommendations.

## 1. Introduction

Respiratory virus infections account for significant morbidity and mortality across the globe [1,2,3,4]. The prevention and control of this group of viruses is challenging due to their high transmissibility and capacity to evolve and cause epidemics and pandemics quickly. This is exemplified by the emergence of two pandemics of respiratory viruses in the last two decades, H1N1 influenza in 2009 and SARS-CoV-2 in 2020. Both viruses managed to spread to almost all parts of the world within a short span of time, mainly due to international travel. After the 2009 pandemic, H1N1 cases and deaths continued to occur every year, with peaks varying by season [5] and other epidemiological factors. From January 2020, due to the emergence and spread of the COVID-19 pandemic, reported influenza cases have declined across the world [6,7]. The actual reduction in influenza virus transmission could be due to COVID-19 related non-pharmaceutical interventions [7] that were implemented globally to curb the pandemic. For example, in India, night and weekend curfews were imposed in several states, public and social gatherings were restricted, wearing a mask in public places was made mandatory, and virtual meetings were emphasized instead of in-person meets. Also, the entire public health machinery was diverted towards enhancing testing for COVID-19. Additionally, the lack of reliable and affordable multiplex diagnostic kits for the detection of respiratory viruses poses a challenge for syndromic testing [8]. The reduced ability of the influenza virus to replicate in the presence of SARS-CoV-2 [7,9,10,11] may be another possible reason for the reduced reporting of cases.

SARS-CoV-2 and influenza virus co-infections were reported from various parts of the world [12,13,14,15,16] and were of particular concern due to reported worsening of clinical signs and symptoms in such cases [17,18]. Besides influenza and other respiratory viruses, studies have also reported co-infection of SARS-CoV-2 with other pathogens, including bacteria and fungi [15,16,19,20]. However, most of these studies have been undertaken in hospital settings [21,22], with limited availability of community-based data.

India initiated testing of influenza viruses in 2004 [23], which was gradually expanded and spread through [5] the network of 131 virus research and diagnostic laboratories (VRDLs) in the country [24]. All VRDLs are linked with the Indian Council of Medical Research (ICMR)–National Institute of Virology (NIV), Pune, India, which is also a World Health Organization–National Influenza Center (WHO–NIC). NIV provides training, technical assistance, reagents to all the VRDLs and collects systematic country-wide data on reported influenza viruses for submission to the WHO FluNet. Since the VRDL network was trained for molecular testing of viruses, it was repurposed for COVID-19 testing during the pandemic; the focus on SARS-CoV-2 testing reduced the ability of the network to test for influenza viruses in India.

COVID-19 and influenza illness both present with a similar clinical picture of ILI/SARI (Influenza Like Illness/Severe Acute Respiratory Infection) and have similar WHO case definitions. In early 2020, the WHO advocated leveraging existing ILI/SARI surveillance mechanisms like GISRS (Global Influenza Surveillance and Reponse System) for COVID-19 surveillance [25], and in November 2020, the WHO suggested integration of COVID-19 and influenza surveillance platforms, with a recommendation to test both pathogens simultaneously, preferably with multiplex PCR (Polymerase Chain Reaction) assays [26]. In line with this, and in view of sporadic reports of high rates of co-infection of SARS-CoV-2 in India [27,28], in July 2021, the ICMR established a pan-India ILI/SARI surveillance network in 22 VRDLs throughout the country. This network is uniquely positioned, as each of the 22 sites is linked with a hospital as well as a defined community catchment area, therefore the surveillance samples are representative of both hospital and community settings. In this paper, we have described the SARS-CoV-2 and influenza co-infection cases detected through this dedicated sentinel surveillance along with their clinical course and outcomes for a period of 7 months (Epidemiological (Epi) Week 27 of 2021 to Epi Week 4 of 2022 + 2 days).

## 2. Materials and Methods

### 2.1. Existing Laboratory Surveillance Network in India

India has a network of 131 VRDLs spread across the country. These laboratories are financially and technically supported by the Department of Health Research, Government of India. VRDLs are located within the existing central and state government medical colleges and have trained staff and infrastructure for serological and molecular diagnosis of 20–25 viruses of public health importance, including respiratory viruses. All VRDLs are linked with the Indian Council of Medical Research (ICMR)–National Institute of Virology (NIV), Pune, India, which is a WHO-National Influenza Center and a WHO SARS-CoV-2 Reference Center.

### 2.2. Selection of Laboratories for the ILI/SARI Surveillance Network

22 VRDLs were selected for inclusion in the ILI/SARI sentinel surveillance network. The selection of laboratories was based on their geographical location and previous experience in molecular diagnostics of respiratory viruses. The laboratories were trained on the requirements of the sentinel surveillance network and provided reagents, and they initiated dual testing of influenza viruses and SARS-CoV-2 from 4 July 2021. The surveillance laboratories underwent Inter-Laboratory-Quality-Control (ILQC) by the NIC at NIV, Pune.

### 2.3. Tiered Structure of the ILI/SARI Surveillance Laboratory Network

The ILI/SARI surveillance network is three-tiered, where each site is mapped to a higher-tier laboratory for training, supervision, mentoring, troubleshooting, and quality control. The bottom tier of 15 testing laboratories is mapped to one of the 6 referral laboratories in the middle tier, which in turn is linked with the WHO-NIC & SARS-CoV-2 Reference Center at ICMR-NIV Pune. Each laboratory (NIV Pune, Referral labs, and testing labs) collects and tests 25 samples per week from consenting patients(15 ILI samples from community settings such as Community Health Centers/Primary Health Centers/Urban Health Centers, and 10 SARI samples from hospital settings).

### 2.4. Diagnostic Multiplex rRTPCR (Real Time Reverse Transcriptase PCR) Kit Used by Laboratories in the Network

Samples are tested by a two-step rRTPCR kit designed by ICMR-NIV, Pune, India, for simultaneous qualitative detection and differentiation of influenza viruses and SARS-CoV-2. The first step of the assay detects virus type (influenza A/influenzaB/SARS-CoV-2), and the second step differentiates between influenza virus subtypes. The assay has an internal control and a cocktail of primers and probes against the target genes: ORF1b for SARS-CoV-2, M1 for influenza A (influenza A H1N1 pdm09 and H3N2), and NS2 for influenza B (Yamagata and Victoria lineages). The assay was subjected to internal and external validation against a well-characterized reference panel. The test was introduced in the field only after satisfactory performance was achieved.

### 2.5. Case Definition for COVID-19/Influenza Co-Infection and Influenza A/B Dual Infection Cases

A COVID-19/influenza co-infection case was defined as an individual fulfilling the WHO case definition of ILI/SARI surveillance (fever ≥ 38 °C and cough, with onset during the last 10 days for ILI, and the added hospitalization criterion for SARI)and testing positive for COVID-19 along with influenza A or B virus. An individual fulfilling the WHO criteria for ILI/SARI and testing positive for influenza A and influenza B virus infection was defined as a case of influenza A/B dual infection.

### 2.6. Data Entry and Analysis

A dedicated ILI/SARI surveillance portal has been developed and hosted at ICMR.All the network laboratories enter demographic, clinical, hospitalization, management, and outcome details of the patients along with laboratory testing results on the portal on a weekly basis [29]. Real-time information on trends of influenza and SARS-CoV-2 activity, lineage-wise positivity, seasonal variations, and un-subtypeable strains is generated for both hospital and community samples.

### 2.7. Retrieval of Demographic and Clinical Data

Co-infection cases were identified from the ICMR influenza portal in real-time, and patients were interviewed via phone calls and hospital visits. The adult patients were questioned in their native language on demographic details, influenza/COVID-19 vaccination status, presence of co-morbidities, symptoms developed, complications, progression of disease, and hospitalization details. For cases involving children, a parent/guardian was interviewed. Co-infection cases were regularly followed up until they recovered or were discharged from the hospital. These interviews also helped to validate the data uploaded on the ILI/SARI portal. Each phone call lasted for 5–6 min.

### 2.8. Period of Reporting

ILI/SARI samples reported on the portal from 4 July 2021 to 31 January 2022 (Epi Week 27 of 2021–Epi week 4(+2 days) of 2022) were considered.

## 3. Results

### 3.1. Samples Tested and Respiratory Pathogens Detected through the ILI/SARI Surveillance Network

**Figure 1:**
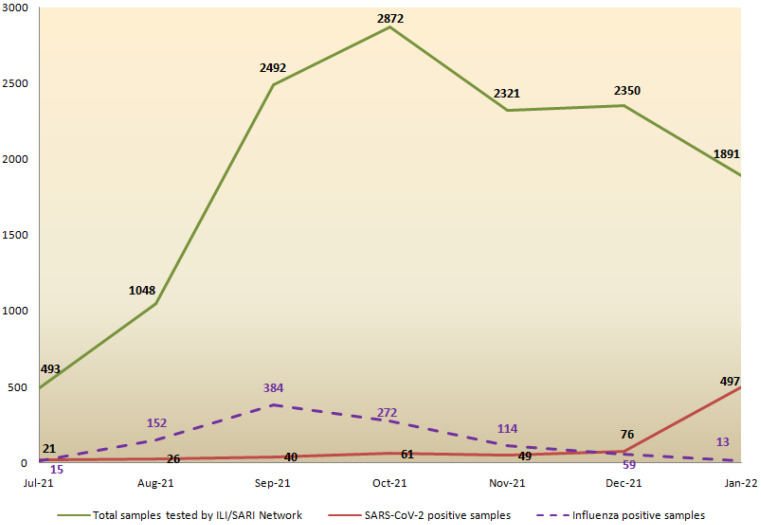
Monthly positivity rate of SARS-CoV-2 and influenza as detected through the ICMR (Indian Council of Medical Research) ILI/SARI (Influenza Like Illness/Severe Acute Respiratory Infection) Surveillance Network.

During the study period (between Epi Week 27 of 2021 and Epi Week 4(+2 days) of 2022), a total of 13,467 ILI/SARI samples were tested by the network, out of which 8776 and 4691 were from community and hospital settings, respectively. A total of 416, 593 and 770 samples were positive for influenza A, B, and SARS-CoV-2, respectively. Monthly influenza and COVID-19 test positivity as captured by the surveillance network is depicted in Figure 1. From July to November 2021, influenza test positivity was higher than COVID-19 positivity, with peak influenza activity observed in September 2021 (384 positive cases out of 2492 samples (15.4%) tested across the network). However, COVID-19 positivity increased in December 2021, with a sharp rise in January 2022 (497 COVID-19 cases out of 1891 samples (26%) tested across the country).

### 3.2. Cases of Co-Infections

A total of 8 cases of co-infection were detected during the study period—5 cases with COVID-19 and influenza co-infection and 3 cases with influenza A/B dual infection. The clinical features, disease progression, and final outcomes of the 5 COVID-19/Influenza co-infection cases are described briefly in Table 1.

The five COVID-19/influenza co-infection cases are described below:

i. Infecting virus type: Two cases had infections with SARS-CoV-2 and influenza A H3N2, while three cases were co-infected with SARS-CoV-2 and influenza B Victoria virus.
ii. Demographic details including co-morbidity: Three of the co-infection cases were children with ages ranging from 1–8 years, while there were 2 adults aged 18 and 74 years. The elderly patient was a known case of COPD (Chronic Obstructive Pulmonary Disease) and CKD (Chronic Kidney Disease) and was on dialysis. The other patients did not have any co-morbidity. Two cases each were reported from the western state of Maharashtra and the northeastern state of Assam, whereas the remaining case was from the southern state of Kerala.
iii. Symptoms, severity, and hospitalization: The symptoms reported by these patients were fever, cough, running nose, and shortness of breath. Two patients were managed at home, and three patients had to be hospitalized.

Brief description of the cases:

Case 1: an eight-year-old child developed fever and dry cough and tested positive for influenza A H3N2 and SARS-CoV-2 three days after onset of symptoms. He was managed at home, and his condition remained stable. Illness resolved within two days with symptomatic management. He did not require administration of oxygen. His childhood immunization schedule was complete as per age.

Case 2: an 18-month-old girl with a previous history of febrile seizures had a fever lasting for 8 h. She was administered oral Paracetamol at home and was hospitalized by her parents within a few hours when the fever did not resolve. She did not develop any respiratory symptoms, and her fever resolved within a day of admission. She was kept under observation and discharged on the third day. She did not require intensive care or oxygen administration, nor did she develop seizures during illness. Her vitals and biochemical parameters remained stable during her hospital stay. No radiographic reports were available. She tested positive for COVID-19 and influenza B Victoria. Her childhood immunization schedule was complete for age.

Case 3: a 1-year-old boy presented with fever, cough, running nose, shortness of breath, and stridor at rest and was hospitalized. He was diagnosed with pneumonia and had to be administered oxygen to maintain saturation at 98%. He was hospitalized for 12 days and discharged after resolution of symptoms. He remained stable during his hospital stay and did not require intensive care. No radiographic reports were available. He tested positive for COVID-19 and influenza B Victoria. His childhood immunization schedule was complete for age.

Case 4: an 18-year-old girl developed fever, cough, and running nose and tested positive for influenza B Victoria and SARS-CoV-2 2 days after onset of symptoms. She was managed at home, and her symptoms resolved within 10 days. She had no known co-morbidity and had not received influenza or COVID-19 vaccine.

Case 5: a 74-year-old male, a known case of COPD and CKD, was admitted with fever, shortness of breath, productive cough, and running nose 2 days after the development of symptoms. He was admitted with the differential diagnoses of lower respiratory tract infection/acute exacerbation of COPD/Acute on chronic kidney disease. On examination, he was conscious, oriented, with a pulse rate of 82/minute, BP 120/70 mm Hg with bilateral lung crepitations and rhonchi. He had deranged renal functions (Urea 185 mg/dL, Creatinine 9.8 mg/L) and hyperkalemia (5.7 mEq/L) and had to undergo dialysis, following which his parameters improved (Urea 123 mg/dL, Creatinine 4 mg/L, Potassium: 4.1 mEq/L). His blood counts remained normal during the hospital stay. His chest X-ray showed bilateral infiltrates with cardiomegaly and pulmonary edema, and chest CT scan findings were suggestive of bronchiectasis. He tested positive for COVID-19 and influenza A H3N2. CORADS scoring was not available. He was administered Cefoperazone and Azithromycin, was hospitalized for 18 days, and discharged on resolution of symptoms.He did not require intensive care or oxygen support. He had not been vaccinated for influenza within the past year, and his COVID-19 vaccination status was unknown.

Outcome: All the co-infection cases were alive on discharge and had resolution of symptoms. None of the three hospitalized patients required ICU (Intensive Care Unit) admission or mechanical ventilation.

The three influenza A/B dual infection cases were from a 6-year-old child from Maharashtra (western India), a 30-year-old male, and a 23-year-old female from Rajasthan (northwestern India). The child had mild symptoms (fever, dry cough, and running nose) for 4–5 days and was managed at home, while both adult patients were hospitalized due to other causes. Both patients were admitted to the same hospital, developed fever, and tested positive during their hospital stay. Both recovered and were discharged after redressal of the primary etiology. All three cases were co-infected with influenza A H3N2 and influenza B Victoria lineage viruses. Much of their clinical history could not be retrieved.

## 4. Discussion

Several studies have reported co-infection of respiratory pathogens along with SARS-CoV-2 [15,16,19,20]. There are also multiple reports of COVID-19 and influenza co-infections globally [12,13,14]. A systematic review and meta-analysis of 26 studies through September 2020 reported the pooled prevalence of COVID-19 and influenza co-infection as 0.8% [30]. Another meta-analysis of 118 co-infection studies through February 2021 reported a pooled prevalence of 19% co-infections and 24% superinfections in COVID-19 cases. A total of 92% of the studies were conducted in hospital settings, and influenza A, B, and Respiratory Syncytial Viruses (RSV) were seen to be the most common co-infecting pathogens [31]. Co-infections of SARS-CoV-2 with influenza virus have been highlighted as a cause of concern due to demonstrated worsening of the clinical picture in such cases. A hospital-based study in Saudi Arabia noted increased mortality and ICU admissionsin influenza A/COVID-19 co-infections [18]. Triple co-infections with both influenza A and B and SARS-CoV-2 have also been reported [32].

In view of the emerging global recommendations to launch comprehensive surveillance programs for other respiratory pathogens alongwith SARS-CoV-2 [33,34] and availability of only limited, sporadic reports of co-infections from India [27,35], concerted efforts were made by the Indian Council of Medical Research to establish pan-India surveillance for SARS-CoV-2 and influenza viruses. One of the objectives of our surveillance program is to understand the proportion of influenza cases occurring along with SARS-CoV-2 and as standalone infections in the backdrop of the ongoing COVID-19 pandemic. Within the 7 month period of our study, we detected only 5 cases of influenza and SARS-CoV-2 co-infection out of the 13,467 ILI/SARI cases tested, thus indicating a low prevalence of co-infections during the reporting period. We identified more influenza B viruses as compared to influenza A, which was associated with severe disease in in-vitro studies and animal models [36,37]. In our study, severity and complications of the disease and hospital admission were associated with extremes of age and existing co-morbidities of the patients rather than the infecting virus strains. No mortality was recorded; however, hospitalization was reported in three patients in extremes of age. Our surveillance has also demonstrated a possible inverse relationship between influenza and SARS-CoV-2 activity. However, this observation may be biased as we have not been able to account for non-pharmaceutical interventions that were implemented during winter 2021 in times of COVID-19 upsurge, leading to a reduction in influenza virus transmission. Since its inception, our network has captured more influenza cases than COVID-19. This may be attributed to the time of initiation of surveillance in July 2021, when the peak of the second wave of COVID-19 in May–June had just subsided, and SARS-CoV-2 transmission was low in India. From July to November 2021, we witnessed an increased influenza activity, peaking in September 2021, which coincided with the monsoon upsurge previously reported from India [5]. However, influenza cases declined in December 2021–January 2022, when COVID-19 cases were again on the rise in the country. Another objective of our surveillance program is to understand the evolution of influenza viruses in the community by continuous and repeated sampling of ILI/SARI patients reported from a defined catchment area. To fulfill this objective, all unsubtypable influenza strains at the sites are referred to the NIC for further characterization.

Our study has limitations. We have presented data of only 7 months from Epi Week 27 as our network was initiated in July 2021; we have initiated testing of only influenza viruses with SARS-CoV-2 and have not been able to include other viruses, particularly RSV; we have not been able to differentiate between co-infection and superinfection cases. Our study has strengths as it reports data from a geographically distributed network of 22 sites from India, which is one of the most populous countries in the world. Besides, most of the studies reported globally have considered co-infections in hospitalized patients, whereas our study reports co-infections in both community and hospitalized patients, with a predominance of community samples. The sensitivity and robustness of our surveillance program are demonstrated by its ability to detect influenza cases when COVID-19 activity was at its peak in December 2021–January 2022. The trends of rise and fall in COVID-19 cases in India, seen in our network, coincide with the national trends [38].

To the best of our knowledge, this is the first report from India on COVID-19/influenza co-infection cases identified through sentinel surveillance from community settings. Our efforts of parallel testing for influenza and SARS-CoV-2 are in line with WHO recommendations of integrated testing for COVID-19 and influenza. We report circulation of influenza viruses in the community as well as hospital settings across the entire country, even during the COVID-19 pandemic, with type B being the dominant influenza virus subtype presently circulating in India. Overall, our study reports a low prevalence of co-infections of SARS-CoV-2 and influenza (0.04%) along with favorable clinical outcomes even in hospitalized patients with pre-existing co-morbidities. As the world is progressing towards COVID-19 endemicity, it is important to strengthen surveillance capacities for other respiratory pathogens, which may lead to significant morbidity and mortality and overburden the health care systems in the future. Adopting cost-effective and reliable testing methods for the diagnosis of respiratory viruses will help in the early detection and prevention of epidemics/pandemics.

## Figures and Tables

**Table 1:** Characteristics of the 5 individuals with COVID-19/influenza co-infection.

Case No.	Region	Age/Gender	Setting	Signs and Symptoms	ILI/SARI	Pathogens	Managed at Home/Hospital	Duration between Onset of Symptom and Hospital Admission	Duration of Hospital Admission	ICU/HDU/CCU Admission	Need of O_2_/Ventilator	Chest CT Scan Findings	Final Outcome	Co-Morbidities	Influenza Vaccination History
1	Western India	8Y/M	Community	Fever, dry cough	ILI	H3N2+SARS-CoV-2	Home	-	-	-	No	NA	Resolved in 2 days	None	Not vaccinated
2	Western India	18m/F	Hospital	Fever	SARI	Victoria+SARS-CoV-2	Hospital	Few hours	3 days	No	No	NA	Discharged in 3 days	None	Not vaccinated
3	North-Eastern India	1Y/M	Hospital	Fever, cough, runny nose, shortness of breath, stridor in calm patient	SARI	Victoria+SARS-CoV-2	Hospital	Few hours	12 days	No	Yes (O_2_)	NA	Discharged after 12 days	None	Not vaccinated
4	North-Eastern India	18Y/F	Community	Fever, cough, runny nose	ILI	Victoria+SARS-CoV-2	Home	-	-	-	No	NA	Resolved in 10 days	None	Not vaccinated
5	Southern India	74Y/M	Hospital	Fever, shortness of breath, runny nose	SARI	H3N2+SARS-CoV-2	Hospital	2 days	18 days	No	No	Few atelectatic bands in lower lobes of both lungs. Fibroatelectatic changes in right lower lobe suggestive of bronchiectasis; mild pulmonary hypertension	Discharged after 18 days	CKD (on dialysis), COPD	Not vaccinated

M = Male, F = Female, Y = Years, m = months, NA = Not available, COPD = Chronic Obstructive Pulmonary Disease, CKD = Chronic Kidney Disease. - = Not applicable, O_2_ = Oxygen, ILI/SARI = Influenza Like Illness/Severe Acute Respiratory Infection, ICU = Intensive Care Unit, HDU = High Dependency Unit, CCU = Critical Care Unit.

## Data Availability

Anonymized datasheet used for analysis is provided as an excel file..
